# Practical Points

**Published:** 1895-03-23

**Authors:** 


### PRACTICAL POINTS.

*Disinfecting House.—Matron asks: I want to know the cheapest and simplest way in which I could have a disinfecting-room for mattresses, &c., for hospital of 50 beds? Could an old mortuary standing away from main building be used as such?*

*Matron.*—There can be no objection to using an old mortuary building as a disinfector-house provided it can be divided by a solid air-tight partition or wall into two parts. The apparatus must be provided with two doors, one to open into one part of the divided building, the other into the other part. The clothes or other things to be disinfected are put in at one door and taken out when finished at the other, and thus infected and disinfected things are kept entirely separate. The apparatus itself to be efficient must be heated by steam, and be provided with a means of drying the air. Lyon's steam disinfector is a good one, but costly. We believe the patent has expired or will shortly expire, and that there are other and less costly apparatus in the market.

